# Combining epidemiology and economics to assess control of a viral endemic animal disease: Porcine Reproductive and Respiratory Syndrome (PRRS)

**DOI:** 10.1371/journal.pone.0274382

**Published:** 2022-09-09

**Authors:** Pablo Valdes-Donoso, Lovell S. Jarvis

**Affiliations:** 1 Department of Clinical Sciences, Faculty of Veterinary Medicine, University of Montreal, St-Hyacinthe, Quebec, Canada; 2 Department of Agriculture and Resource Economics, University of California Davis, Davis, California, United States of America; King Abdulaziz City for Science and Technology (KACST), SAUDI ARABIA

## Abstract

Porcine reproductive and respiratory syndrome (PRRS) is an extremely contagious disease that causes great damage to the U.S. pork industry. PRRS is not subject to official control in the U.S., but most producers adopt control strategies, including vaccination. However, the PRRS virus mutates frequently, facilitating its ability to infect even vaccinated animals. In this paper we analyze how increased vaccination on sow farms reduces PRRS losses and when vaccination is profitable. We develop a SIR model to simulate the spread of an outbreak between and within swine farms located in a region of Minnesota. Then, we estimate economic losses due to PRRS and calculate the benefits of vaccination. We find that increased vaccination of sow farms increases the private profitability of vaccination, and also transmits positive externalities to farms that do not vaccinate. Although vaccination reduces industry losses, a low to moderate vaccine efficacy implies that large PRRS losses remain, even on vaccinated farms. Our approach provides useful insight into the dynamics of an endemic animal disease and the benefits of different vaccination regimens.

## Introduction

Animal diseases generate economic losses by limiting production and increasing costs for disease control [1, 2]. Incentives to control animal diseases are usually related to the nature and severity of diseases, the effect of diseases on human health and/or trade, the type of animal production system, and government policies [3]. Success in controlling animal diseases typically depends on measures implemented by farmers and their neighbors. Farmers have incentives to protect their own animals from disease and those actions often reduce the risk that animals on other farms will contract a disease. In some cases, governments mandate or subsidize private disease control actions to promote social benefits. Farmers may also engage in collective actions (e.g., sharing sanitary information or specific disease control strategies). Few economic studies have analyzed the potential benefits of collective disease control actions [1].

Porcine Reproductive and Respiratory Syndrome (PRRS), which emerged in the 1980s and is now one of the costliest swine diseases in the US, offers an opportunity to study the possible benefits of individual and collective control strategies. PRRS increases mortality in sows and piglets, decreases reproduction in sows, and decreases feed conversion in feeder pigs [4]. Holtkamp et al. (2013) estimated PRRS caused annual losses in the US swine industry of more than $660 million in 2005–2010 [5]. PRRS is caused by a virus that is highly infectious, resistant to cold temperatures and highly mutagenic [6]. Endemic strains appear to have low virulence, generating outbreaks with relatively low mortality and morbidity losses. Nonetheless, major outbreaks occur almost every year, usually associated with emergent viral strains that cause high mortality and morbidity. During such outbreaks, multiple genetically-related viral strains may be identified in a region or even within a single farm [7]. Because vaccines can only target a few known viral strains, commercial vaccines may confer only partial protection against emergent fields strains [7–9].

PRRS does not harm humans and is endemic to most swine-producing countries. Outbreaks thus do not invoke international trade restrictions. In the US, PRRS is a non-reportable disease, and the government does not collect data on outbreaks or play a regulatory role. Hence, PRRS control currently depends wholly on farmers’ voluntary decisions to adopt control measures. Most farmers have implemented biosecurity procedures, a few have invested in bio-filters, and some in vaccination [4]. Some farmers have created voluntary regional control programs (RCPs) to encourage sharing PRRS status and control strategies [10]. Nonetheless, PRRS continues to cause large losses.

PRRS may resemble, in many aspects, other viral diseases that affect animal or human populations. It spreads within and between farms (i.e., confined animal populations) via the movement of sick animals, contaminated fomites, and airborne transmission [4]. In the US, swine production facilities hold large numbers of densely confined animals and are often connected via commercial links. Once an animal is infected, PRRS virus spreads quickly through different sections of the initial facility and it may remain in the facility for more than half a year [10]. It may also move to other facilities.

Mathematical models can be used to simulate the spread of crop, animal, or human diseases, to shed light on disease dynamics and analyze the effects of different control strategies [11]. For example, Tardy et al. (2022) used an agent-based spatial model to investigate how host distribution and habitat fragmentation affect the dynamics of tick-borne diseases. The authors concluded that the host settlement strategy and the proportion of habitat available to hosts determined the super-spread of infected ticks, which could be relevant to designing public health interventions to control tick-borne diseases [12]. Although the dynamics of the disease are complex, various studies on COVID-19 diffusion modeling have been published to help design public health strategies [13–17]. For example, Kumar et al. (2021) used a compartmental mathematical model to simulate and predict the spread of COVID-19 in Argentina, obtaining results and offering useful insights that might help authorities design more effective control strategies [17].

Different studies utilize models to simulate the spread of infectious diseases in livestock and explore the effect of different control strategies, e.g., [18–20]. Some studies evaluate the economic cost of a disease and the net benefits of specific control investments or policy interventions. For example, Bicknell et al. (1999) built a bioeconomic model to simulate the dynamics of bovine tuberculosis (bTB) as it was transferred from a wild animal, the possum, to cattle populations in New Zealand. They assessed different control strategies and concluded that the prevailing control policies decreased bTB prevalence, but undesirably removed some private incentives to control bTB [21].

In this study, we build a novel SIR model that simulates a PRRS spread between and within farms in an RCP in Minnesota by using farm characteristics, animal movements, and farm spatial location. We estimate the production losses from infected and dead animals on each farm and incorporate the decision to use a vaccine to control PRRS. We then quantify the private benefits and the externalities created by vaccination and use the results to make judgements about the profitability of vaccination and their implications for public policy.

## Materials and methods

### Data source

We use data from 817 swine farms located in the RCP-N212 region of Minnesota. For each farm, we know the type of animal produced, the animal inventory and the expected animal movements between swine farms (taken from [22]). Farm types are characterized by the animals produced, e.g., sow farms deliver 2-3-week-old weaned pigs weighing 10-20lb. to fattening or finishing farms, which raise pigs for about 16–20 weeks until they reach market weight (225-300lb.). In some cases, nursery farms act as an intermediate stage between sow farms and fattening farms for 6–7 weeks. Boar-stud farms supply semen to sow farms [23]. Table 1 summarizes the number of farms of each type, the average animal inventory on each type of farm, and total number of animals in our farm sample.

**Table 1:** Inventory distribution per farm type in the RCP-N212 region.

Farm Type	No. Farms	Farm Avg. Inventory	Total Animals
Boar Stud Farms	8	158 (86)	1,260
Fattening Farms	537	1,984 (1,692)	1,065,666
Nursery Farms	83	3,637 (3,728)	301,893
Sow Farms	189	1,247 (1,774)	235,593
Total	817		1,604,412

*Note*: Standard deviations of animal inventory are in parenthesis. Inventory and total number of animals in sow farms include only sows.

### Model structure

**Fig 1:**
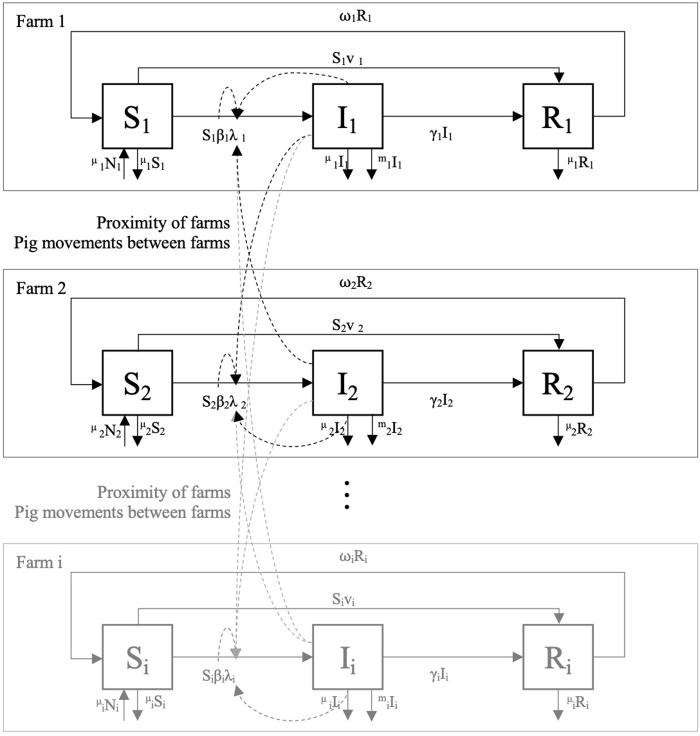
Conceptual flow of our SIR model.

We use a SIR model with a typical structure in which animals fall into three disease categories, susceptible (S), infected (I), and recovered (R) during each week (the time unit) of the 52-week simulated period (Fig 1). Our model assumes the probability of disease transmission from one farm to another is heterogenous, i.e., transmission is a function of farm type, farm proximity to other farms and expected animal movements between swine farms. We derive the parameters defining the rates of transmission from one farm to another, and from one animal to another within a farm (once infected), from the scientific literature. We modify all parameters in the scenarios when vaccination occurs, again based on the scientific literature. Sow vaccination changes the probabilities that the sow farm will be infected and that it will infect other farms if it becomes infected. Note that vaccination reduces mortality, the effects of morbidity, and the amount of virus that an animal sheds, even if an animal is infected.

We begin each simulation at week 0 with ten pigs infected in the same farm (named the index case) while animals on all other farms in the RCP-N212 are 100% PRRS negative (virus-free). As the virus spreads, we determine the number of infected ($I_{i_{t}}$) and dead animals ($mI_{i_{t}}$) in each farm (*i*) each week (*t*) throughout 52 weeks (one year). In each week, a fraction of infected animals dies due to PRRS at rate *m* and another fraction recovers at rate γ, defined as $\gamma =\frac{1}{D_{\phi }}$, where *D*_*ϕ*_ is the length of infection (number of weeks), and the subscript *ø* stands for a farm that either has sows or non-sows.

Since infection does not provide long-lasting immunity, recovered animals (R) may transfer to S again at rate ω. Recovered individuals return to the susceptible status at rate $\omega =\frac{1}{DI}$, where *DI* is the duration of immunity post-infection. When a sow farm vaccinates, we assume that a fraction (*v*) of susceptible sows (S) flows to the recovered category (R). Such a fraction is given by the vaccine efficacy (we assume that 100% of the sows are vaccinated once a farm implements vaccination), where 1-*v* is the fraction of sows for which the vaccination failed to provide immunity.

For simplicity, we assume that farm re-stocking rates are identical to their exit rates (μ), e.g., farms restock when they ship fat pigs to slaughter or when animals die. Therefore, the number of animals on each farm decreases in our model only temporarily (one week) via the mortality caused by PRRS virus. We use the differential Eqs 1, 2 and 3 to estimate the number of animals within each farm, in each category (i.e., S, I, R, or dead):

$$\frac{dS_{i}}{dt}=\mu N_{i}-S_{i}\beta_{i}N_{i}^{-1}\sum_{j}\rho_{ij}I_{j}+\omega_{i}R_{i}-\mu S_{i}-vS_{i}$$ (1)

$$\frac{dI_{i}}{dt}=S_{i}\beta_{i}N_{i}^{-1}\sum_{j}\rho_{ij}I_{j}-\gamma_{i}I_{i}-\mu I_{i}-m_{i}I_{i}$$ (2)

$$\frac{dR_{i}}{dt}=\gamma_{i}I_{i}-\omega_{i}R_{i}-\mu R_{i}+vS_{i}$$ (3)

The term $\beta_{i}N_{i}^{-1}\sum_{j}\rho_{ij}I_{j}$ represents the rate at which susceptible animals on farm *i* become infected, commonly referred to as the force of infection ($\lambda_{i}=\beta_{i}N_{i}^{-1}\sum_{j}\rho_{ij}I_{j}$). *λ*_*i*_ depends on a disease transmission parameter (*β*_*i*_) and on all pairwise interactions (*ρ*_*ij*_) between susceptible (*i*) and infected farms (*j*). The disease transmission parameter $\beta_{i}=R_{0_{i}}*\frac{1}{D_{\phi }}+R_{0_{i}}*\mu$, is the per capita rate of effective contact between an infected and a susceptible animal. *R*_*0i*_ is the basic reproductive ratio or the number of secondary cases originated from primary a case [11]. *D*_*ø*_ is the length of PRRS infection depending on the type of animal (i.e., sows or pigs).

The force of infection in a farm is subject to all possible connections (via distance and animal movements) with all farms within the RCP-N212 region. Thus, in each time unit, we calculate *ρ*_*ij*_ between all possible pairs of farms in the region. Eq 4 shows the estimation of the pairwise interaction term:

$$\rho_{ij}(K,L)=K_{ij}+L_{ij}-K_{ij}L_{ij}$$ (4)

*ρ*_*ij*_ states that the probability of infection depends on the distance (*K*_*ij*_) and the movements of animals (*L*_*ij*_), if any, from farm *j* to *i*. Eq 5 denotes that the probability of disease transmission given that the distance between a pair of farms (*d*_*ij*_) exponentially decays following a Poisson distribution, i.e., *K* ~ *Pois*(*λ* = *E*(*K*)),

$$K_{ij}=e^{\beta_{0}+\beta_{1}d_{ij}}$$ (5)

Eq 6 stands for the weekly probability of disease transmission due to animal movements from a farm *j* to a farm *i*. If animal movements are likely to occur between a pair of farms, then *L*_*ij*_ *= L*_*ij*_, and *L*_*ij*_ *=* 0 otherwise (See [22] for details in predicting pig movements between farms in the RCP-N212 region). *L*_*ij*_ depends on the prevalence (*p*) of PRRS in a given animal movement from an infected farm *j*, the average number of animals transported in each movement (*n*_*ij*_), and the total annual number of movements (*m*_*ij*_) from a farm *j* to a farm *i*.


$$L_{ij}=(1-{\left(1-p\right)}^{n_{ij}})\frac{m_{ij}}{52}$$ (6)

Table 2 summarizes the parameters collected from the epidemiological literature that we use in Eqs 1 through 6. The transmission of PRRS between and within farms differs for each type of farm, using different basic reproductive ratios (R_0_), lengths of the duration of infection (D) and immunity (DI), and rates of mortalities. Transmission also depends on the different probabilities of infection, given the distance between farms (see Eq 5) and the nature of animal movements (see Eq 6). To simulate the effects of low (endemic) and high (emergent) virulent virus strains, we use the minimum and maximum values of parameters from Table 2, except for DI, where we use min and max value inversely. For probability of infection from a source of infection, we use the upper and lower bounds of the 95% confidence intervals from Eq 5. Recognizing that an outbreak might consist of both endemic and emergent varieties, we also simulate results using the average values of the parameters in Table 2 and the fitted K_ij_, instead of the CIs, from Eq 5. The impact of PRRS may vary greatly depending on a series of characteristics that involve the agent, host, and environment.

**Table 2:** Parameters used in the model.

Parameter	Avg. (Min, Max)	Units	Source
Basic reproduction number in sows (*R*_*0s*_)	2.12 (0.14, 3.22)	-	[24]
Basic reproduction number in pigs (*R*_*0p*_)	2.57 (1.80, 3.30)	-	[25]
Duration of infection in unvaccinated sows (*D*_*S*_)	4 (1, 6)	Wks.	[26]
Duration of infection in unvaccinated pigs (*D*_*P*_)	8 (4, 12)	Wks.	[24]
Duration of infection in vaccinated sows (*D*_*Sv*_)	2.5 (0.7, 4.2)	Wks.	[27]
Duration of immunity (*DI*)	18 (16, 20)	Wks.	[28, 29]
Mortality rate in sows (m_s_)	0.0035 (0.0002–0.0083)	Animals/Wk.	[30–32]
Mortality rate in boars (m_b_)	0.0001 (0–0.0002)	Animals/Wk.	Assumed
Mortality rate in nursery pigs (m_n_)	0.021 (0.0012–0.0583)	Animals/Wk.	[31–33]
Mortality rate in fattening pigs (m_p_)	0.0044 (0.0003–0.0167)	Animals/Wk.	[31–33]
PRRS prevalence in positive lot	0.6 (0.3, 0.9)	-	Assumed
Exit and entry rate (μ)	0.001	-	Assumed
Vaccine efficacy (*Ve*)	0.2, 0.5, 0.8	-	Assumed
Probability of infection from a source of infection at 0, 2.3, 4.6, 4.7, 6.6, and 9.1 km.^a^	1, 0.018, 0.009, 0.013, 0.009, 0.009	-	[34, 35]

^a^ Values used to fit Eq 5.

### Estimation of losses under the baseline scenario

No information currently exists regarding the expected virulence of a PRRS strain (or set of strains) during an outbreak. Lacking such information, we hypothesize three different viral strains, a low virulence strain that represents endemic virus strains, a high virulence strain to represent emergent strains, and an average virulence strain. We use parameters from Table 2 to calculate the spread of PRRS due to these three different viral strains under the initial assumption that no farm in the region adopts vaccination to control the disease. In these simulations, we estimate the weekly (*t*) mortality and morbidity (productivity) losses on each farm *i*. We translate these “physical” losses into economic losses by multiplying the number of dead animals ($mI_{i_{t}}$) by their market price (*P*_*i*_) and the number of infected pigs ($I_{i_{t}}$) by the decrease in present value of their expected future production (*V*_*i*_) [36]. Table 3 shows the per head prices (P) and the per-animal decrease in value (*V*) used to calculate the economic losses from mortality and decreased performance (reproduction and feed conversion), respectively. For simplicity, we assume that each farm only has pigs of one type (e.g., finishing farms have only feeder pigs and sow farms have only sows). We use the average values of V and P to estimate farm losses because we do not have detailed data on the weight or age distribution of different cohorts within farms. Eq 7 shows the calculation of the yearly total losses in each farm (*i*), which are the sum of the total mortality losses ($P_{i}\int_{0}^{t}mI_{i_{t}}dt$) and the total morbidity losses ($V_{i}\int_{0}^{t}I_{i_{t}}dt$).


$$TL_{i}=P_{i}\int_{0}^{t}mI_{i_{t}}\;dt+V_{i}\int_{0}^{t}I_{i_{t}}\;dt$$ (7)

**Table 3:** Economic loss (USD) due to mortality or morbidity per type of animal.

Associated Cost	Type of Animal ^a^	Avg. (Min, Max)	Description	Source
Mortality (*P*)	Sows	42 (31, 54)	Market price of live sows.	[37]
	Nursery pigs	25 (10, 40)	Market price of pigs 10–40 lb.	[37]
	Feeder pigs, Hogs	46 (10, 82)	Market price for pigs > 40 lb.	[37]
	Boars	30 (17, 44)	Price of live animal basis	[37]
Morbidity ^b^ (*V*)	Sows	3 (1.60, 4.90)	Decrease in wean pigs’ production	[10, 38]
	Nursery pigs	0.30	Decrease of conversion rates	[33]
	Feeder pigs, Hogs	0.30	Decrease of conversion rates	[33]
	Boars	133 (127, 138)	Doses of semen discarded	[39, 40]

^a^ Sows are confined in sow farms, nursery pigs in nursey farms, feeder pigs in finishing farms, and boars in boar stud farms.

^b^ Decrease in Production Per Infected Animal-Week

### Estimations of cost and benefits under vaccination

We assume a fraction of the susceptible population (S) moves to the recovery stage (R) once a farm is vaccinated. Following the vaccine manufacturers’ indications, we assume a farm vaccinates three times a year. The vaccinated fraction that moves from S to R is given by the vaccine efficacy (ve). As the actual efficacy of vaccines is unknown, we assume three values to explore the effect of changing efficacy on our results (ve = 0.2, 0.5, 0.8). For simplicity, we assume that vaccinating farms vaccinate 100% of sows. We modify the recovery rate (*γ*) to $\gamma_{i}^{V}=\frac{1}{(1-0.3)D_{\phi }}$, assuming vaccination reduces the duration of infection by 30% [27]. For those farms that do not vaccinate, there is no direct transit of animals from S to R, and the recovery rate remains as it is (i.e., $\gamma_{i}=\frac{1}{D_{\phi }}$). Changing these assumptions allows us to simulate how vaccination affects the diffusion of a PRRS outbreak and the resulting mortality and morbidity caused on all farms.

We estimate the private gross benefits of vaccination (*B*_*i*_) by comparing the change in losses caused by PRRS in a vaccination scenario to the losses caused by PRRS in the baseline scenario. Eq 8 shows the gross benefits per animal for each vaccinating farm. The numerator of the right side of Eq 8 represents the difference between the losses that occur on a farm *i* when no control strategies are adopted (i.e., under baseline scenario = *Base*) versus the losses when the same farm vaccinates (i.e., under vaccination = *Vac*) in a year. The denominator (*q*_*i*_) is the number of animals on a farm *i*. The subscript *k* refers to the vaccination scenario, so *B*_*ik*_ varies between farms and within farms given a different vaccination strategy (e.g., individual vaccination versus different levels of collective vaccination).


$$PMB_{ik}=\frac{P_{i}\left(\int_{0}^{t}mI_{i_{t}}\;dt_{Base}-\int_{0}^{t}mI_{i_{t}}\;dt_{Vac}\right)+V_{i}\left(\int_{0}^{t}I_{i_{t}}\;dt_{Base}-\int_{0}^{t}I_{i_{t}}\;dt_{Vac}\right)}{q_{i}}$$ (8)

Though we know that vaccination of one sow farm may have external effects on other farms, we ignore these effects initially, simulating our model 189 times, once for each sow farm, assuming that only one sow farm is vaccinating in each simulation. While unrealistic, these simulations provide a first approximation of the effects of vaccination on individual farms, demonstrating that the benefits of vaccination may vary across sow farms, e.g., depending on farm location, links with other farms, farm’s characteristics. Having used Eq 8 to estimate the benefits from individual vaccination, we then use it to simulate the effects of collective vaccination in sow farms, assuming successively the vaccination on 25%, 50%, 75% and 100% of sow farms. The sow farms grouped into the first 25% vaccinated are those with the highest estimated benefits from vaccination, as determined by the simulations when only one farm vaccinates. Groupings for 50%, 75% and 100% of sow farms followed the same approach.

## Results

### Estimation of losses under the baseline scenario

**Fig 2:**
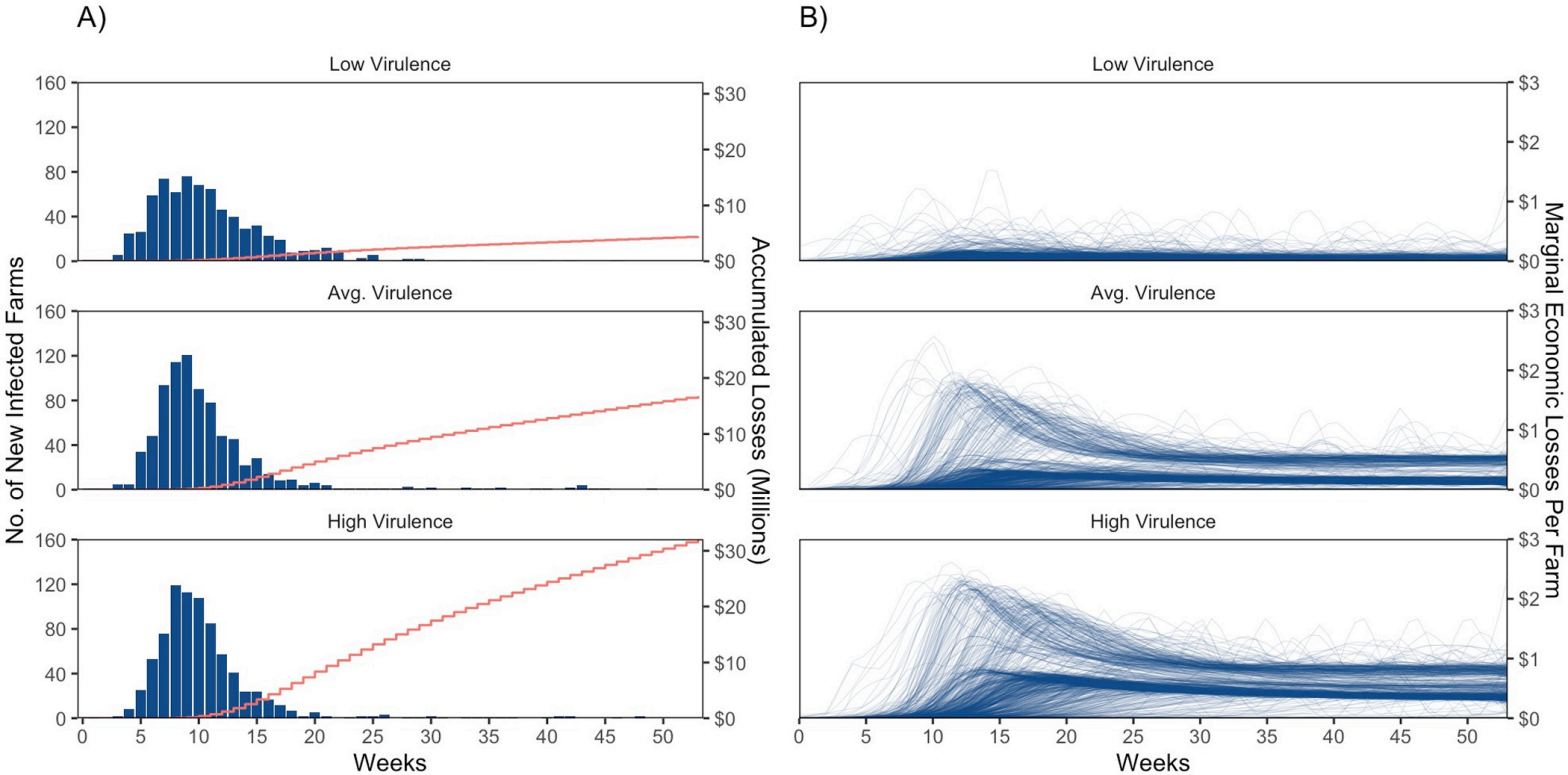
Baseline scenario of PRRS spread under three strains. (A) Number of New Positive Farms (Bars) and Cumulative Economic Losses (Red Lines). (B) Marginal Economic Losses for Each Infected Farm over Time.

As baseline scenarios, we simulate losses from a low virulent PRRS strain, a high virulent strain, and a strain of average virulence, assuming that no farm in the RCP-N212 region adopts vaccination or any other control strategy. For each level of virulence, we estimate the economic losses. Fig 2A shows our estimates for the number of farms newly infected each week (aka disease incidence) and the related accumulated losses (red line) during an uncontrolled outbreak caused by three different strains. When no farms attempt to control PRRS, an outbreak caused by each of the three simulated strains infects a large proportion of farms. However, losses from a strain with high virulence are more than seven times higher than those caused by a strain with low virulence. If caused by a low virulent strain, the number of newly infected farms peaks in the 9^th^ week (incidence of 9%) and total losses in the 52^nd^ week surpass $4 million. If caused by a high virulent strain, the number of newly infected farms peaks in the 8^th^ week, (incidence of 15%), and total economic losses are about $32 million by the 52^nd^ week and still rising (Fig 2A).

Fig 2B shows the weekly marginal damage curves for individual farms over the 52-week period under both low and high virulent strains. Given that each farm is identifiable, we observe that individual farms suffer varying levels of weekly damage after being infected, i.e., the timing and the magnitude of damages differ across farms even when affected by the same strain (Fig 2B). Our model simulations indicate that different types and numbers of animals within each farm, farms with more close neighbors, and farms with more connections suffer more marginal losses. Our results resonate with previous epidemiology literature that account for the relationship of PRRS transmission given the density of farms in an area [4, 35, 41], the movement of sick animals [4, 22], and the maintenance of the disease among groups of pigs within farms [4, 10]. Our simulations indicate that the damages from an infection also continue for many weeks, consistent with the results from a previous study that found losses from a PRRS infection on sow farms decreased production for at least 35 weeks [10].

Table 4 presents the simulated economic losses inflicted by a low, average, and high virulence strain, respectively, on different farm types, if a PRRS outbreak occurs when farmers do not invest in disease control. Total losses vary greatly by the type of strain expected, being $4 million, $17 million, and $32 million, respectively. Losses from low virulence strains are primarily from morbidity (production losses), but the larger losses from higher virulence strains come increasingly from mortality. Finishing and nursery farms always suffer the largest aggregate losses because they have, in the aggregate, 80% of animals in the region (Table 1). However, aggregate damages on sow farms increase rapidly as the infecting strain becomes more virulent. Note that nursery and finishing farms do not vaccinate animals because the expected losses per animal are too low to make vaccination profitable. Boar stud farms cannot market semen from infected or vaccinated animals (as tests cannot discriminate between the two) and thus also do not vaccinate.

**Table 4:** Estimated losses from an uncontrolled PRRS outbreak (*USD millions*), by viral strain and farm type.

Strain	Farm Type	Productivity Losses	Mortality Losses	Total Losses
**Low Virulence**	Boar Stud	1.2	0.0	1.2
	Finishing	2.0	0.3	2.3
	Nursery	0.4	0.1	0.5
	Sow	0.4	0.1	0.5
	***Total***	***4*.*0***	***0*.*4***	***4*.*4***
**Avg. Virulence**	Boar Stud	2.3	0.0	2.3
	Finishing	3.9	2.6	6.5
	Nursery	0.7	1.3	2.0
	Sow	5.7	0.3	6.0
	***Total***	***12*.*6***	***4*.*2***	***16*.*8***
**High Virulence**	Boar Stud	3.1	0.0	3.1
	Finishing	4.5	11.4	15.9
	Nursery	0.7	3.2	3.9
	Sow	8.3	0.9	9.2
	***Total***	***16*.*6***	***15*.*5***	***32*.*1***

Note: these results are for what we refer to as the baseline scenario.

**Fig 3:**
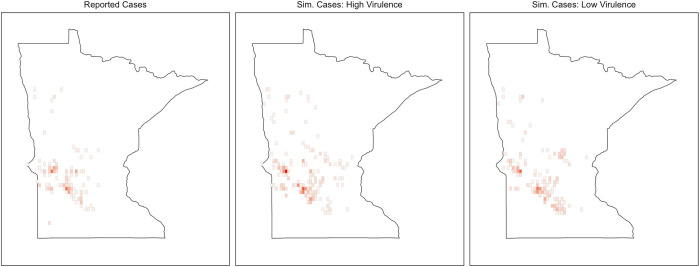
Spatial distribution of reported PRRS cases (2012–2014), and simulated PRRS cases for a high and a low virulent strain.

The simulated spatial distributions of infected farms, whether caused by a high or a low virulence strain, closely mimic the spatial distribution of the actual outbreak between 2012 and 2014, as reported by RCP-N212 participants [41]. This is a partial, if somewhat informal, indication that our models behave plausibly (Fig 3).

**Fig 4:**
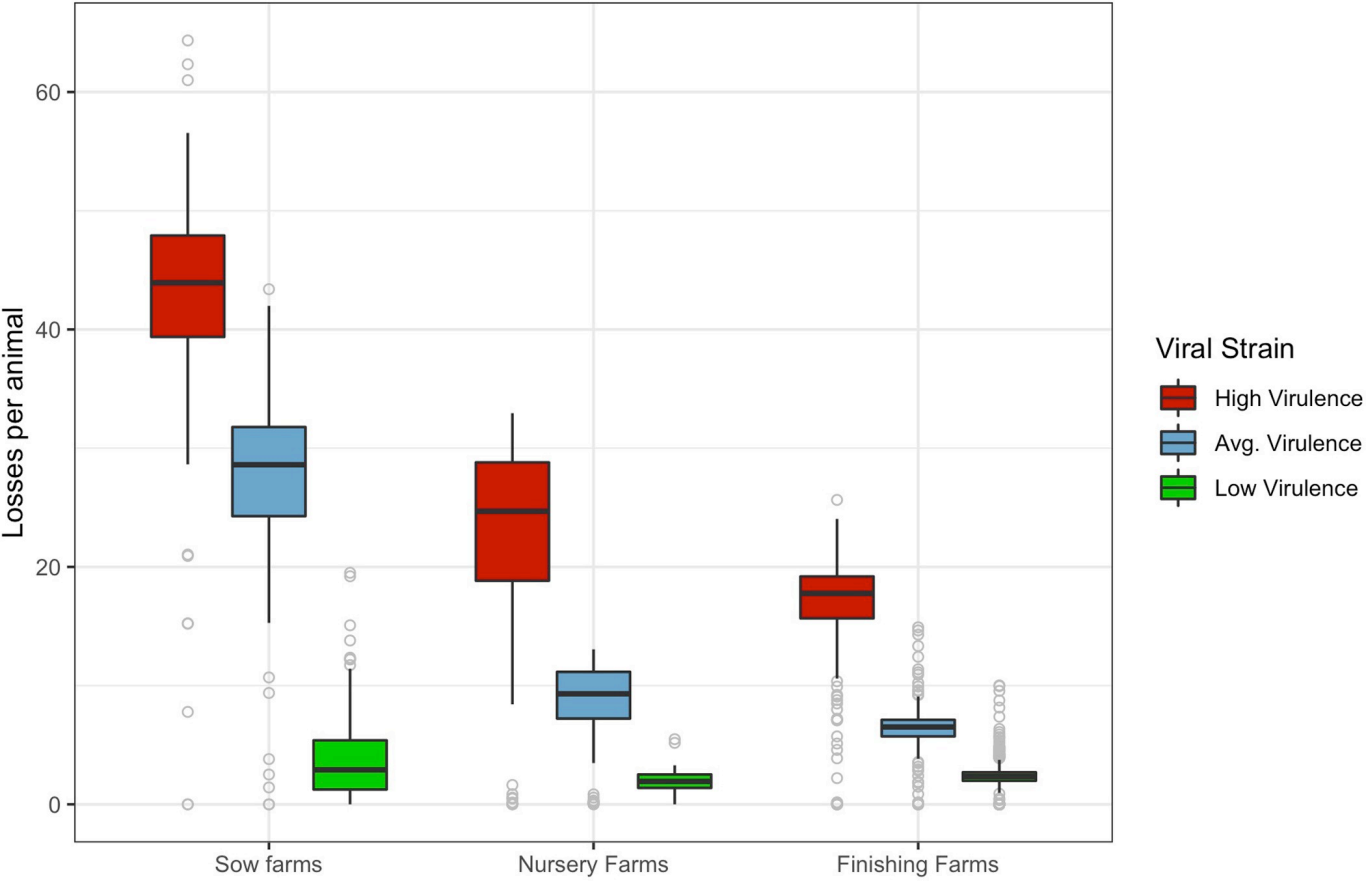
Distribution of losses per animal estimated under two viral strains in sow, nursery, and finishing farms.

Fig 4 shows the estimated damages per animal (i.e., total losses divided by the total number of animals in the farm) on different types of farms and differentiated by infecting strain. For all strains, the per-animal losses are highest in boar stud farms (per-animal losses far exceed $100), followed by losses on sow, finishing, and nursery farms. The high expected losses per sow in the “average” scenario is consistent with the observation that some sow farms vaccinate to control PRRS, while the low value of per-animal losses on finishing and nursery farms are consistent with the observation that they do not vaccinate (Fig 4).

### Estimations of costs and benefits under vaccination

According to current research, PRRS vaccination is only partly effective in protecting animals from infection [8]. Commercial vaccines perform better against homologous challenges (strains genetically close to those for which the vaccine has been created), than against heterologous challenges (strains with high genetic variability). However, vaccination appears to reduce clinical symptoms, virus shedding, and the intensity and duration of outbreaks, even when animals are infected. Thus, vaccination may help to mitigate the spread and effects of an outbreak even if does not prevent infection.

In several figures below, we present the estimated marginal benefits for each vaccinating farm under different vaccination scenarios. The marginal benefits are calculated by subtracting the losses caused by PRRS under vaccination from the losses caused by PRRS in the baseline scenario. We use $5.9/year as the marginal cost per vaccinated sow, calculated from a retail vaccine cost of about $1.5 per dose (three doses per year), plus 30% for logistics, labor, and other costs. Vaccination is profitable on those farms where the marginal benefits surpass the marginal cost of vaccination.

**Fig 5:**
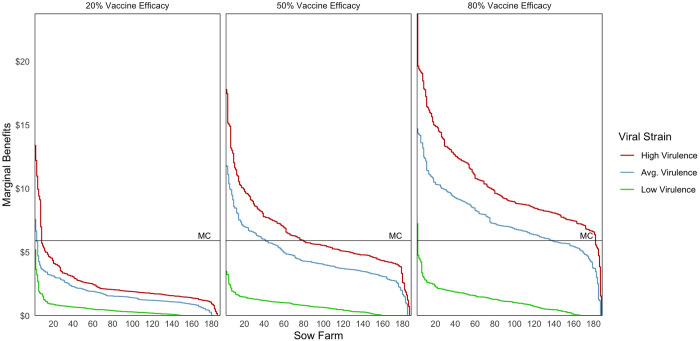
Marginal benefits from individual adoption of vaccination when varying infecting strains and vaccine efficacy. Note: The estimated marginal cost of vaccination (MC) is a constant $5.9/year per animal.

Fig 5 shows the marginal benefits of vaccination on sow farms when vaccination occurs only on one sow farm of the region at a time. These simulations depict the expected reduction in net losses from PRRS for low, average, and high virulent strains for vaccines varying between 20%, 50% and 80% efficacy. We order farms from highest to lowest estimated gross benefits from vaccination showing clearly that farms are likely to obtain different benefits from vaccination and thus are likely to have a different willingness to vaccinate. Our simulations also indicate that the benefits from individual vaccination depend importantly on the infecting strain and on vaccine efficacy. When the infecting strain is of low virulence, the net benefit of vaccination is positive for only one farm and then only if vaccine efficacy is high. However, when the infecting strain has higher virulence, vaccination appears profitable on many sow farms and nearly all when the vaccine has 80% efficacy.

As our model is interactive, the risk of infection on many farms and the private profitability of vaccination usually depends on whether other farms are also vaccinating. Given the large number of sow farms, it was infeasible to explore this issue in detail. However, we explore this effect more generally by estimating the private benefits on all sow farms as an increasing number of sow farms are assumed to vaccinate collectively (i.e., 25%, 50%, 75% and 100% of sow farms vaccinating).

**Fig 6:**
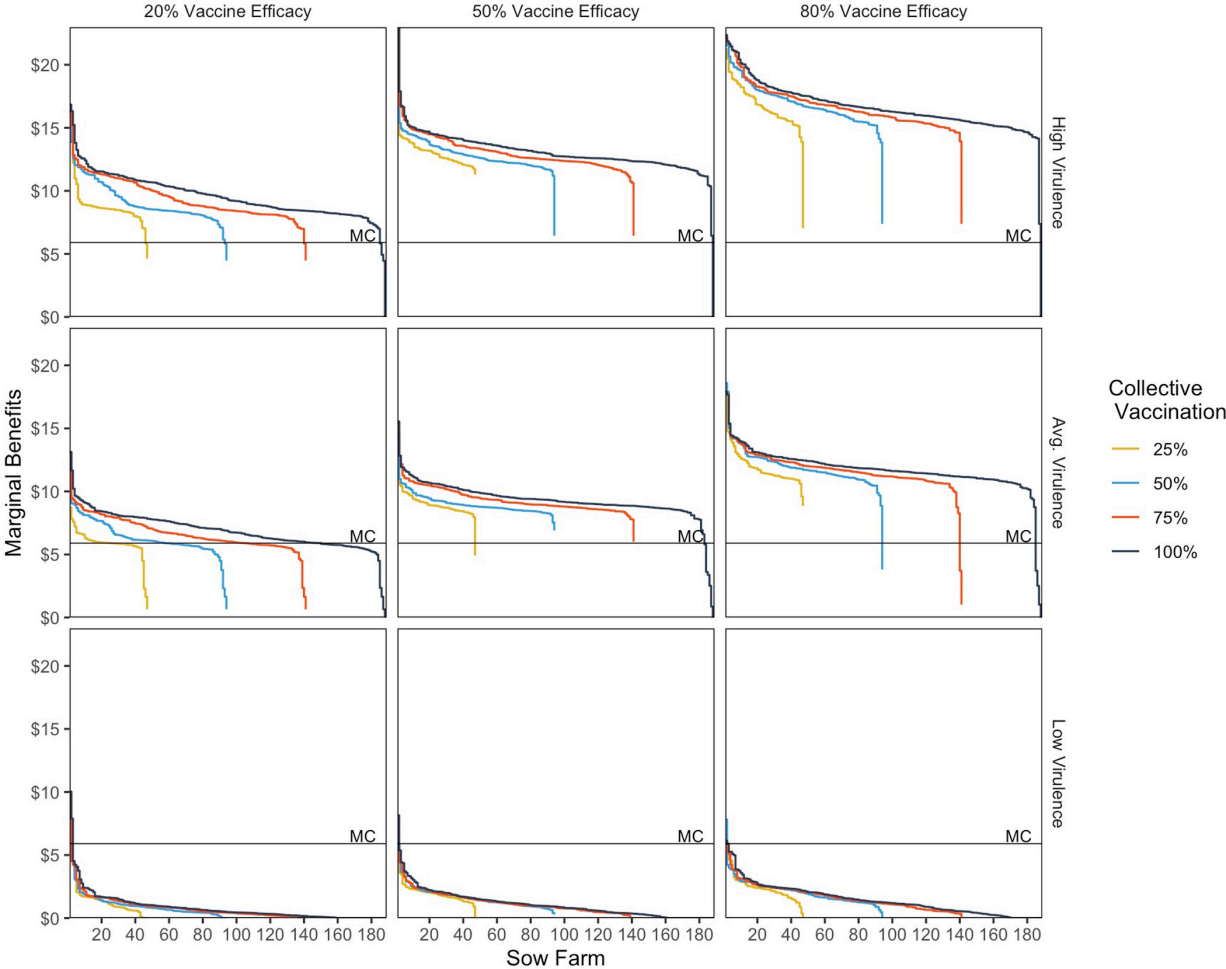
Marginal benefits of vaccination for three infecting strains, increasing amounts of collective vaccination and varying vaccine efficacy. Note: The estimated marginal cost of vaccination (MC) is a constant $5.90/year per animal.

Fig 6 shows the results for the three virulence strains (low, average, and high) and three levels of vaccine efficacy. For purposes of exposition, we rank sow farms on the x-axis from the highest to lowest private profitability of vaccination within each collective vaccination group in order to show more clearly how rising rates of collective vaccination affect the profitability of vaccination for each of the farm cohorts vaccinated.

The results are striking, even if anticipated. The profitability of vaccination increases as vaccine efficacy increases and as the infecting strain is more virulent. When the infecting strain is of low virulence, vaccination is privately profitable for very few sow farms. However, when the infecting strain has average or high virulence, vaccination is profitable for a large majority of sow farms, even when vaccine efficacy is only 50%.

Fig 6 also shows that the marginal benefits of vaccination for first 25% of farms rises almost monotonically as more farms vaccinate, and this effect continues for subsequent cohorts. Although increasing the amount of collective vaccination generally increases the estimated profitability of vaccination for individual farms, benefits fall slightly on some farms.

Greater collective vaccination makes vaccination profitable for a successively larger fraction of farms. For example, with a strain of average virulence, as the number of farms vaccinating rises to 25%, 50%, 75%, and 100%, vaccination is profitable for 5%, 59%, 73%, and 79%, respectively, of the farms vaccinating, even when the vaccine has only 20% efficacy. With a strain of high virulence and 50% efficacy, vaccination is privately profitable for nearly all farms when all are vaccinating.

We had anticipated that vaccinating a higher number of farms would reduce disease risk for all and, as the risk of disease fell, the profitability of vaccination for individual farms might decline, including on those farms that had vaccinated first. The opposite occurred. We believe collective vaccination has strong positive benefits across farms mainly because vaccination lowers the amount of virus shedding and thus reduces losses for nearly all farms. However, it does not seem likely that this this effect would be perceived by farms acting independently. Some type of collective action might be needed to achieve such positive results.

Three effects are worth summarizing. First, as an additional cohort is vaccinated, e.g., 50% instead of 25% of farms, vaccination is almost always privately profitable for the newly vaccinating farms. Moreover, the private profitability of vaccination for these farms is higher than it was when we assumed these farms vaccinated alone. Second, vaccination of an additional cohort increases the private profitability of vaccination for farms in the prior cohort(s) that were already vaccinating. Third, vaccination appears profitable on nearly all sow farms when all farms are vaccinating if strain virulence is average or high. Vaccination is not privately profitable for several sow farms if vaccine efficacy is low (Fig 6).

Given that increasing vaccination appears to achieve broad benefits, what can be done if vaccination is not privately profitable on all farms? How serious is this problem? We have shown that if virus virulence is average or high, nearly all farms should find vaccination at least marginally profitable. However, vaccination remains privately unprofitable for between 1% and 5% of sow farms even in these favorable situations. Thus, if farms vaccinate voluntarily, we anticipate that some sow farms would not vaccinate and, within a dynamic framework, their decisions not to vaccinate might cause other farms not to vaccinate. Additionally, other farms might find benefits too small to motivate them to implement vaccination, particularly given the likelihood that the expected virus faced might be of low rather than high or average virulence. As a result, it does not appear that voluntary decisions are likely to lead to nearly universal vaccination on sow farms.

Would it be desirable from a social or collective viewpoint to require vaccination on all sow farms even if some farms will lose? How many farms would lose because of mandatory vaccination and how much would they lose? Are the externalities from vaccination sufficiently large to influence public policy decisions? Table 5 contains our estimates of the private and external benefits obtained for different levels of collective vaccination on sow farms, given an average virus strain and different levels of vaccine efficacy. To obtain the net benefits on each farm type, we multiply the number of animals on each farm in our sample by the estimated per-animal benefit (or loss) it gains as vaccination occurs. Total net benefits will vary across farms because farms differ in size (number of animals), type of animals, and estimated benefits (or losses) per animal.

**Table 5:** Private, social, and total net benefits (*US$ Thousands*) from vaccination as sow farm vaccination increases if the expected virus is of average virulence.

Vaccine Efficacy	Percent of Sow Farms Vaccinating	25%	50%	75%	100%
20%	Cost of Vaccination ^a^	398	937	1,238	1,390
	Net Benefits				
	Private Benefits to Sow Farms	-5	89	155	181
	Externalities in Sow Farms	0.3	0.1	0.1	0.0
	Externalities in Non-Sow Farms	90	92	94	95
	***Total Net Benefits***	***85***	***181***	***249***	***277***
50%	Net Benefits				
	Private Benefits to Sow Farms	227	514	723	789
	Externalities in Sow Farms	4	6	3	0
	Externalities in Non-Sow Farms	142	212	274	291
	***Total Net Benefits***	***374***	***732***	***1*,*001***	***1*,*080***
80%	Net Benefits				
	Private Benefits to Sow Farms	403	951	1,239	1,366
	Externalities in Sow Farms	4	12	8	0
	Externalities in Non-Sow Farms	184	387	457	497
	***Total Net Benefits***	***591***	***1*,*349***	***1*,*704***	***1*,*862***

^a^ Cost of vaccination is on a per sow basis. As sow numbers differ across farms, total vaccination costs are not necessarily equal for each quartile of vaccinating sow farms.

Assuming an outbreak of average virulence and vaccine efficacy of 50%, vaccination of the first cohort (25%) of sow farms achieves aggregate net private benefits on sow farms of about $227,000, or about of $4,815 per farm. (See the results for low and high virulent strains in S1 Table). Nonetheless, given the variation in benefits per animal and in farm size, the estimated net private benefits vary greatly among farms (see Fig 6). The average net private benefits decrease slightly (to about $4,200 per sow farm) if all sow farms vaccinate because those who vaccinate later have lower average net benefits. However, expected private benefits from vaccination roughly double if a high instead of an average virulent strain is expected. Alternatively, if farmers vaccinate but experience an outbreak with a low virulent strain, the average loss per farm is about $6,500 (S1 Table).

As noted earlier, vaccination generates important external benefits to non-vaccinating farms. These external benefits rise with higher virulence strains, higher vaccine efficacy and a higher proportion of vaccinated sow farms and they are always substantial in the aggregate. When the expected virus is of average or high virulence, the expected external benefits are considerably larger than the potential losses experienced by the few vaccinating sow farms for which vaccination is unprofitable. However, if the expected virus is of low virulence, the total expected net benefits from sow-farm vaccination are always negative (S1 Table).

The results of our model suggest that the expected economic benefits for individual farms from vaccination are highly sensitive to the decision of other farmers to vaccinate, the vaccine’s efficacy, and the virulence of the infecting strain. Moreover, while vaccination reduces losses on sow farms, the losses expected from a PRRS outbreak remain very large even with vaccination by 100% of sow farms. For example, when vaccine efficacy is 50% and the infecting strain is of average virulence, vaccination reduces total sow farm losses and total swine sector losses only about 13% and 6%, respectively (see Tables 4 and 5). Our model indicates that vaccination is a useful control instrument and probably should be expanded. Nonetheless, given results from our model, large losses from PRRS would remain even if all sow farms vaccinate.

## Discussion

PRRS causes great economic damage and is now an endemic disease in the US. PRRS virus mutates frequently, and the emergent virus strains are often highly virulent and associated with very damaging outbreaks. PRRS is non-reportable in the US and its control depends exclusively on the voluntary actions taken individually and/or collectively by producers. In this study, we sought to shed light on the differences among producers in the willingness to vaccinate against PRRS and whether vaccination might be more profitable if a larger number of sow farms were to vaccinate. We also sought to determine the size of positive externalities on other farms from rising sow farm vaccination.

We use scientific data to specify a SIR model with heterogenous PRRS transmission rates that depend on the type of animal produced, geographical location, and commercial connections (i.e., animal movements). We use the model to simulate losses caused by disease and then explore how vaccination is expected to reduce disease damage on individual farms. No information exists regarding the expected distribution of virus strains during an outbreak [42]. Similarly, commercial vaccines are known to be more effective against known endemic strains than against emergent strains. However, having no definitive information regarding vaccine efficacy against different strains, we explore the effects of vaccination assuming different levels of vaccine efficacy on three hypothetical strains. In each vaccinating farm, we estimate the reduction in damages associated with vaccination as the gross benefits from vaccination. Subtracting the estimated cost of vaccination from the gross benefits, we obtain estimates of the expected net benefits on each vaccinating farm. By varying the assumed share of sow farms that are vaccinating in different simulations, we also analyze how the private profitability of vaccination changes as sow vaccination is expanded to additional farms. We are also able to estimate positive externalities perceived by non-vaccinating farms.

Our results provide important insights into PRRS outbreaks and their control. For example, expected damages from an outbreak associated with a low virulence virus are relatively small compared with the expected damages from outbreaks associated with either a high virulence strain or even an average strain are much higher. Sow farms vary in their expected losses from a PRRS outbreak. In general, farms that are geographically located closer to other swine production facilities and/or that engage in more animal movements to and from swine producing facilities have greater losses. These results are expected as they are built into the model transmission parameters. However, these parameters are taken from previous scientific studies and placing them within the SIR model allows us to grasp better their aggregate implications. The per farm estimates of expected damages also have potential value to help individual producers decide whether vaccination is desirable (because of confidentiality limitations, we cannot divulge our estimates for individual farms, but this obstacle might be overcome in future studies).

The expected private profitability of vaccination also varies across sow farms, rising sharply as virus virulence increases, as vaccine efficacy rises, and as more sow farms vaccinate. Vaccination is only profitable for a few sow farms when an outbreak is caused by a low virulence strain, regardless of vaccine efficacy or the share of sow farms vaccinating. However, vaccination is profitable for most sow farms even if they are the only farm vaccinating when the expected outbreak is caused by a high or average virulence strain. Increasing vaccine efficacy also increased the profitability of vaccination when the expected virus virulence is high or average but was of little importance when the expected virus virulence is low. Increasing the share of sow farms that vaccinate had a positive impact on the profitability of vaccination across essentially all sow farms. We examined the effect of vaccination by 25%, 50%, 75% and 100% of sow farms. When 50% of sow farms vaccinated, the profitability of vaccination on the 25% of farms that was previously vaccinating actually increased and this effect continued as the share of farms vaccinating increased to 75% and to 100%. The result is worth emphasizing. Our model estimates that nearly all sow farms will find vaccination profitable if the expected virus virulence is high or average, if vaccine efficacy is at least 50%, and if all sow farms vaccinate. We believe that increasing vaccination increases its marginal benefits because vaccination reduces the number of animals infected and also the amount of virus shedding among infected animals, leading to less contagion and lower damages within the region.

Finding out that vaccinating more farms has positive advantages for all is difficult to determine if producers are vaccinating one by one. In a world in which vaccination is voluntary, some farms will not vaccinate. Vaccination is not profitable on some farms and on some other farms the expected benefits from vaccination are positive, but probably too low to motivate vaccination. Additionally, since producers face considerable uncertainty regarding the expected virulence of any virus challenge in the next period, risk avoidance will probably reduce vaccination even more. If so, the number of farms vaccinating in equilibrium may be much less than 100%. There is some confirmation of this conjecture in that the Morrison Swine Health Monitoring Program (MSHMP) reports that about 30% of the roughly 700 sow farms it surveys in the US are vaccinated against PRRS [43]. Of course, improved and/or cheaper vaccines could increase this equilibrium, as could the diffusion of more information about the potential benefits from vaccination on individual farms. Theoretically, a considerably more complex model that takes account of each producer’s optimal choices re vaccination, given other producers optimal choices, could estimate directly an equilibrium.

The damages in feeder pigs are too low to justify vaccinating these animals. However, vaccination on sow farms conveys significant benefits to non-sow farms. Vaccination on sow farms reduces infections throughout the region, not just sow farms. In the simulations involving an average virus strain, with variation in vaccine efficacy and 100% of sow farms vaccinating, non-sow farms receive 27% to 34% of the total benefits from vaccination. These external benefits are significantly larger than the estimated losses that would accrue to a few vaccinating sow farms if vaccination was mandatory. However, compensating such farms would be administratively difficult.

Our SIR model estimates that an uncontrolled PRRS outbreak with a virus of average virulence would cause about $17 million in total damages to the 817 swine farms in this region of Minnesota, assuming no vaccination. These farms have about 1.6 million sows and pigs. To be comparable with published inventories, we could include newly born piglets on sow farms of roughly 0.3 million animals. Extrapolating and assuming 75 million swine in the US [44], our local estimates an uncontrolled outbreak nationally could cause about $790 million in production and mortality losses. This figure is somewhat larger than the estimate by Holtkamp et al. (2013) of PRRS losses of $660 million [5], but the assumptions in the models are similar, but not identical. National swine numbers have increased since 2013, so we might anticipate higher damage figures. Our estimates of total PRRS losses roughly double if we assume an expected virus with high rather than average virulence. Thus, it is possible that PRRS losses are significantly larger than previously estimated.

Our model estimates that vaccination is profitable for many sow farms, and would reduce damages from PRRS on non-sow farms. Nonetheless, sow farm vaccination is not a powerful instrument to control PRRS. Assuming an expected virus of average virulence, 50% vaccine efficacy, and 100% of the sow farms vaccinated, vaccination reduces total PRRS losses by roughly $1 million, or 6% of the losses that would occur under the same circumstances except for no sow farm vaccination. These results suggest that vaccination cannot decisively reduce PRRS damage unless the vaccine used has much greater efficacy. Of course, swine producers are also using biosecurity measures and biofilters, but these too appear thus far to have had only limited effect. We had hoped to study the effect of these other control measures, but a lack of data precluded our ability to quantify costs and benefits in any reliable manner.

Finding that current vaccines are unlikely to substantially reduce PRRS losses is disappointing. The result also emphasizes that lack of an effective vaccine leaves us vulnerable when confronted with a highly contagious, pathological disease. Vaccine efficacy is measured by the vaccine’s ability to prevent infection. However, vaccines that reduce the severity of disease, including death, are highly valuable even if they do not prevent infection. We need measures of vaccine effectiveness that take account of the vaccines ability to reduce infection and to reduce disease severity. A PRRS vaccine that does not reduce infection but that converts a lethal disease into a mild disease would be a boon for swine producers.

The choice of parameter values is crucial to model performance. Deterministic and stochastic models use parameters that can be specified or drawn randomly from distributions to assess how variation in the parameters affects the results [11]. We experimented with both types and decided to use fixed parameters. The quality of our estimates depends on the parameter specifications chosen and the accuracy of the prices used to value physical losses. We chose parameters carefully from the epidemiological literature and prices from market records, but others might choose different values. Moreover, as additional scientific information becomes available, parameters could be chosen more accurately, producing improved estimates.

Our sample contains only farms within the RCP-N212 region. In making our estimates, the farms on the outer edge of this geographical area appear more isolated from other swine farms than they actually are. Other farms, just outside the area considered, could be geographically close and thus affect disease risk. Moreover, we do not consider animal movements between the farms in our sample and farms outside the sample because we do not have such information. Lack of such knowledge distorts the results, but we cannot determine by how much. However, such knowledge would almost certainly increase the risk of disease exposure for *edge* farms, and thus increase both expected damages from a PRRS outbreak and also the estimated benefits of vaccination.

We consider our approach as a proof of concept. We believe that our relatively simple SIR model combining epidemiological and economic elements is parsimonious in terms of parameter needs, computationally feasible and provides useful results and insights. A similar approach might be used to study other control strategies for PRRS investments, as well as other diseases in swine or other production systems.

## Supporting information

S1 Table Private, social, and total net benefits (*US$ Thousands*) from vaccination as sow farm vaccination increases if the expected virus is of low or high virulence. (DOCX)

S1 Data (XLSX)
