# Supplementary material for: Combining epidemiology and economics to assess control of a viral endemic animal disease: Porcine Reproductive and Respiratory Syndrome (PRRS)
Source: PLoS One. 2022 Sep 9;17(9):e0274382. doi: 10.1371/journal.pone.0274382 (PMC9462702; doi:10.1371/journal.pone.0274382)
Supplement: S1 Table — (DOCX) [file pone.0274382.s001.docx]

**S1 Table: Private, social, and total net benefits (*US$ Thousands*) from vaccination as sow farm vaccination increases if the expected virus is of low or high virulence.**

| **Strain** | **Vaccine Efficacy** | **Percent of Sow Farms Vaccinating** | **25%** | **50%** | **75%** | **100%** |
| --- | --- | --- | --- | --- | --- | --- |
|  |  | Cost of Vaccination ^a^ | 398 | 937 | 1,238 | 1,390 |
| Low Virulence | 20% | Net Benefits |  |  |  |  |
|  |  | Private Benefits to Sow Farms | -91 | -435 | -974 | -1296 |
|  |  | Externalities in Sow Farms | 3.3 | 2.2 | 1.0 | 0.0 |
|  |  | Externalities in Non-Sow Farms | 38 | 39 | 40 | 49 |
|  |  | Total Net Benefits | -50 | -393 | -932 | -1,247 |
|  | 50% | Net Benefits |  |  |  |  |
|  |  | Private Benefits to Sow Farms | -89 | -350 | -889 | -1,228 |
|  |  | Externalities in Sow Farms | 4 | 4 | 2 | 0 |
|  |  | Externalities in Non-Sow Farms | 38 | 42 | 53 | 63 |
|  |  | Total Net Benefits | -47 | -304 | -834 | -1,165 |
|  | 80% | Net Benefits |  |  |  |  |
|  |  | Private Benefits to Sow Farms | -89 | -337 | -843 | -1,169 |
|  |  | Externalities in Sow Farms | 4 | 5 | 2 | 0 |
|  |  | Externalities in Non-Sow Farms | 39 | 49 | 67 | 78 |
|  |  | Total Net Benefits | -46 | -284 | -775 | -1,090 |
| High Virulence | 20% | Net Benefits |  |  |  |  |
|  |  | Private Benefits to Sow Farms | 147 | 495 | 707 | 813 |
|  |  | Externalities in Sow Farms | 0.4 | 0.4 | 0.3 | 0.0 |
|  |  | Externalities in Non-Sow Farms | 679 | 532 | 518 | 507 |
|  |  | Total Net Benefits | 826 | 1,027 | 1,225 | 1,320 |
|  | 50% | Net Benefits |  |  |  |  |
|  |  | Private Benefits to Sow Farms | 481 | 1,157 | 1,490 | 1,672 |
|  |  | Externalities in Sow Farms | 3 | 8 | 2 | 0 |
|  |  | Externalities in Non-Sow Farms | 729 | 983 | 1,084 | 1,132 |
|  |  | Total Net Benefits | 1,213 | 2,148 | 2,576 | 2,804 |
|  | 80% | Net Benefits |  |  |  |  |
|  |  | Private Benefits to Sow Farms | 751 | 1,744 | 2,285 | 2,529 |
|  |  | Externalities in Sow Farms | 2 | 23 | 15 | 0 |
|  |  | Externalities in Non-Sow Farms | 791 | 1,445 | 1,655 | 1,749 |
|  |  | Total Net Benefits | 1,545 | 3,212 | 3,955 | 4,278 |

^a^ We calculate the cost of vaccination on a per sow basis. Not all sow farms have the same number of sows, so vaccination costs for the first 25% are not equivalent to the second, third and fourth quartiles of vaccinating sow farms.
